# Schizophrenia mediating the effect of smoking phenotypes on antisocial behavior: A Mendelian randomization analysis

**DOI:** 10.1111/cns.14430

**Published:** 2023-08-31

**Authors:** Minghui Zhang, Jie Tang, Wei Li, Kaizhong Xue, Zirui Wang, Yayuan Chen, Qiang Xu, Dan Zhu, Mengjing Cai, Juanwei Ma, Jia Yao, Yijing Zhang, He Wang, Feng Liu, Lining Guo

**Affiliations:** ^1^ Department of Ultrasound Tianjin Medical University General Hospital Airport Hospital Tianjin China; ^2^ Department of Radiology and Tianjin Key Laboratory of Functional Imaging Tianjin Medical University General Hospital Tianjin China; ^3^ Department of Radiology Tianjin Medical University Cancer Institute and Hospital, National Clinical Research Center for Cancer, Key Laboratory of Cancer Prevention and Therapy, Tianjin, Tianjin's Clinical Research Center for Cancer Tianjin China; ^4^ Department of Radiology Tianjin Medical University General Hospital Airport Hospital Tianjin China

**Keywords:** causality, mediating effect, schizophrenia, smoking

## Abstract

**Aims:**

Previous studies have indicated that smoking is linked to an increased risk of developing schizophrenia, and that individuals with schizophrenia are more prone to engaging in antisocial behavior. However, the causal effects of smoking behaviors on antisocial behavior and the potential mediating role of schizophrenia remains largely unclear.

**Methods:**

In the present study, using the summary data from genome wide association studies of smoking phenotypes (*N* = 323,386–805,431), schizophrenia (*Ncases* = 53,386, *Ncontrols* = 77,258), and antisocial behavior (*N* = 85,359), we assessed bidirectional causality between smoking phenotypes and schizophrenia by the Mendelian randomization (MR) approach. Using a two‐step MR approach, we further examined whether causal effects of smoking phenotypes/schizophrenia on antisocial behavior were mediated by schizophrenia/smoking phenotypes.

**Results:**

The results showed that smoking initiation (SmkInit) and age of smoking initiation (AgeSmk) causally increase the risk of schizophrenia (SmkInit: OR = 2.06, 95% CI = 1.77–2.39, *p* = 4.36 × 10^−21^; AgeSmk: OR = 0.32, 95% CI = 0.16–0.62, *p* = 8.11 × 10^−4^, Bonferroni corrected). However, there was no causal effect that liability to schizophrenia leads to smoking phenotypes. MR evidence also revealed causal influences of SmkInit and the amount smoked (CigDay) on antisocial behavior (SmkInit: OR = 1.28, 95% CI = 1.17–1.41, *p* = 2.53 × 10^−7^; CigDay: OR = 1.16, 95% CI = 1.06–1.27, *p* = 1.60 × 10^−3^, Bonferroni corrected). Furthermore, the mediation analysis indicated that the relationship between SmkInit and antisocial behavior was partly mediated by schizophrenia (mediated proportion = 6.92%, 95% CI = 0.004–0.03, *p* = 9.66 × 10^−3^).

**Conclusions:**

These results provide compelling evidence for taking smoking interventions as a prevention strategy for schizophrenia and its related antisocial behavior.

## Graphical abstract

This study showed that the relationship between smoking initiation and antisocial behavior was partly mediated by schizophrenia using a two‐step Mendelian randomization analysis.

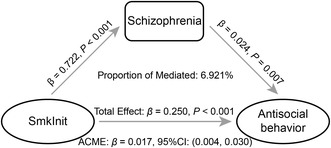

## 1 INTRODUCTION

Smoking continues to be the most significant preventable cause of death in our society, and it may be an independent risk factor for schizophrenia.^1^ The excessive smoking behavior of individuals with psychiatric illnesses, particularly schizophrenia, has been widely recognized for many years.^2^ In fact, the prevalence of smoking among people with schizophrenia is at least two to three times higher than that of the general population.^3^ Currently, the mechanism underlying the high‐smoking prevalence among individuals with schizophrenia remains unclear. Three possible explanations have been proposed for this association: (1) aspects of the illness itself may lead to smoking behavior among patients; (2) smoking could be an additional risk factor for developing schizophrenia; or (3) genetic and/or environmental factors may contribute to both the development of schizophrenia and smoking behavior.^4^, ^5^ In addition, heavy smokers are more common in schizophrenia, and individuals with schizophrenia are significantly less likely to quit smoking compared to both the general population and those with other psychiatric disorders.^4^, ^6^ Besides, individuals with schizophrenia also tend to start smoking at a younger age, with most patients initiating smoking during their teenage years, before the onset of illness.^7^

There are several lines of evidence that schizophrenia increases the risk for aggressive behavior.^8^, ^9^ Generally, two main factors contribute to violence in patients with schizophrenia: a history of conduct disorder and antisocial personality disorder, as well as the psychopathology of acute schizophrenia.^10^ While aggression or violence can sometimes be justified, such as in maintaining public order or enforcing the law, antisocial behavior can also include aggressive and violent acts. This could potentially explain the higher rates of violent crime observed in patients with schizophrenia.^11^ Despite the notable link between schizophrenia and violence, schizophrenia accounts for less than 10% of the overall violence in society. It has been consistently demonstrated that the presence of comorbid substance abuse among individuals with schizophrenia significantly amplifies the probability of violent behavior compared to those without such comorbidity.^12^, ^13^, ^14^ Tobacco products contain the addictive drug nicotine, as well as numerous toxic chemicals, making them a common substance of abuse. Nicotine dependence is a chronic and relapsing disease due to its addictive properties and unpleasant withdrawal symptoms. While both tobacco use and schizophrenia have been linked to antisocial behavior,^15^ the causal pathway of smoking, schizophrenia, and antisocial behavior remains unclear.

Mendelian randomization (MR) is a statistical approach that utilizes the measured variation in genes of known function to examine the causal effect of a modifiable exposure on an outcome.^16^ One advantage of MR is that it is capable of overcoming the challenge of confounding and reverse causality that may arise between the exposure and outcome.^17^ In recent years, MR has become a widely used approach due to the expansion of large‐scale genome‐wide association studies (GWASs), which have provided a wealth of genetic data for research studies. To date, previous MR studies have suggested a causal relationship between smoking initiation and schizophrenia.^18^, ^19^, ^20^, ^21^ However, the causal effect of schizophrenia liability on smoking initiation was inconsistent in previous studies.^18^, ^19^, ^21^ Furthermore, no studies have investigated the bidirectional causal associations between schizophrenia and other smoking phenotypes, such as smoking cessation, amount smoked, and age of smoking initiation.

In the current study, we aimed to use the MR approach to investigate the bidirectional causal effects of smoking phenotypes (including smoking initiation, smoking cessation, amount smoked, and age of smoking initiation) on schizophrenia. In addition, we sought to explore the causal relationships between smoking phenotypes and antisocial behavior, and if so, whether schizophrenia can serve as a mediator.

## 2 METHODS

### 2.1 Study design

The current MR analysis is based on three key assumptions: (1) the genetic variants used as instrumental variables (IVs) should be strongly associated with exposure; (2) the genetic variants used as IVs should not be associated with any confounding factors that could affect the association between exposure and outcome; (3) the genetic variants should only affect the risk of the outcome through exposure. In this study, we performed the MR analysis using publicly available GWAS summary datasets of European descent, including four smoking phenotypes, schizophrenia, and antisocial behavior.^22^, ^23^, ^24^ Specifically, smoking phenotypes included smoking initiation (SmkInit), defined as a binary phenotype where smokers were individuals who reported having ever smoked regularly; smoking cessation (SmkCes), which contrasted current versus former smokers; amount smoked (CigDay), which was measured as the number of cigarettes smoked per day among current and former regular smokers; age of smoking initiation (AgeSmk) was defined as the age at which the individual began smoking regularly.

In the current study, bidirectional two‐sample MR analyses were first performed to assess the causal relationship between smoking phenotypes and schizophrenia. Then, two‐sample MR analyses were performed to assess the causal effect of smoking phenotypes/schizophrenia on antisocial behavior. Finally, two‐step MR analyses were performed to examine the mediation effects of schizophrenia/smoking phenotypes on the association between smoking phenotypes/schizophrenia and antisocial behavior.

### 2.2 Data sources and genetic instruments

The GWAS summary data for smoking phenotypes, namely SmkInit, SmkCes, CigDay, and AgeSmk, were obtained from the study that included GWAS summary statistics for 3.4 million individuals from four major clines of global ancestry.^22^ The GWAS summary data for schizophrenia were obtained from a recent study, which included up to 76,755 cases and 243,649 controls.^23^ The schizophrenia samples were collected from various studies on schizophrenia. The diagnostic procedures used in these studies were similar, and the final diagnosis was based on a best‐estimate consensus lifetime diagnosis.^23^ The diagnostic criteria utilized for making the diagnoses were in accordance with either the Diagnostic and Statistical Manual of Mental Disorders, Fourth Edition (DSM‐IV) or the International Classification of Diseases, 10th Revision (ICD‐10). These widely accepted diagnostic manuals provide standardized criteria for the diagnosis of schizophrenia and ensure consistency in the diagnostic process across studies. Only the summary statistics of European ancestry samples in smoking phenotypes and schizophrenia were selected to construct genetic IVs (805,431 individuals for SmkInit, 388,313 individuals for SmkCes, 326,497 individuals for CigDay, and 323,386 individuals for AgeSmk; 53,386 cases and 77,258 controls for GWAS summary data of schizophrenia). Summary statistics for antisocial behavior were obtained from a meta‐analysis of data from 28 discovery samples (*N* = 85,359) where all the participants were of European ancestry.^24^ The diagnosis of antisocial behavior in the GWAS dataset used in our study varied across different samples due to the utilization of multiple cohorts. As the GWAS data were derived from multiple studies with their own unique diagnostic processes, there may be some variation in the specific diagnostic criteria used for identifying antisocial behavior, and please refer to the corresponding literature for detailed diagnostic criteria.^24^ All these included original studies claimed to have been approved by local ethics committees, and informed consent was obtained from all the participants. The data sources used in the MR analyses are summarized in Table S1 and the participant selection procedure is depicted in Figure S1.

We selected the IVs at the genome‐wide significance threshold (*p* < 5 × 10^−8^) from the GWAS summary data. To select independent variants, LD clumping was conducted based on *r* ^2^ > 0.001 within a 10,000 kb window. If there were no SNPs for exposure in the outcome datasets, we replaced them with their proxy SNPs (*r* ^2^ > 0.8), defined using 1000 Genomes European sample data. We then harmonized the effect alleles of these variants in both the exposure and outcome datasets. After data harmonization, we removed palindromic SNPs with intermediate allele frequencies, and outlier pleiotropic SNPs detected by the heterogeneity test (*p* < 0.05) were discarded using RadialMR.^25^ We also performed Steiger filtering to remove any SNPs that explained more variance in the outcome than the exposure.^26^ The remaining SNPs were used for MR analysis.

### 2.3 MR analyses

Bidirectional MR analyses were carried out to examine the reciprocal causal links between smoking phenotypes and schizophrenia. In addition, we conducted two‐sample MR analyses to evaluate the causal effect of smoking behaviors/schizophrenia on antisocial behavior. To obtain the standardized effect size and standard error estimates for the GWAS meta‐analysis of antisocial behavior, we utilized the previously described method which involves minor allele frequency and sample size as parameters in the following equation^27^:

$$\beta =\frac{Z}{\sqrt{2P\times \left(1-\mathrm{MAF}\right)\times \left(N+Z^{2}\right)}},\;\mathrm{se}=\frac{1}{\sqrt{2P\times \left(1-\text{PMAF}\right)\times \left(N+Z^{2}\right)}}$$

where *Z* can be calculated as *β*/*se* from the original summary data, *MAF* is the minor allele frequency, and *N* is the total sample size. The standardized effect size and standard error were used for subsequent MR analysis.

The inverse variance weighted (IVW) method was used as the primary method due to its ability to provide accurate estimations.^28^ However, since the IVW method might be affected by invalid IVs and pleiotropy,^29^ we thus conducted four other MR methods, including MR robust adjusted profile score (MR‐RAPS), MR‐Egger, weighted‐median, and weighted‐mode methods, as they make different assumptions regarding the nature of pleiotropy. Specifically, the MR‐RAPS method accounts for systematic and idiosyncratic pleiotropy by incorporating overdispersion and robust loss functions, which can provide a robust inference for MR analysis with many weak instruments^30^; the MR‐Egger method allows all variants to have pleiotropic effects and can provide a consistent estimate of the causal effect under a weaker instrument strength independent of direct effects assumption^31^; the weighted median approach can yield a consistent estimate if at least 50% of weight is derived from valid IVs^29^; the weighted‐mode method forms clusters of SNPs based on their causal effect estimates and estimates the causal effect in the largest cluster of SNPs, thus providing an unbiased estimate if the SNPs contributing to the largest cluster are valid.^32^ Bonferroni correction was performed for multiple testing across all MR tests, and thus the significant *p* value threshold was set at *p*_IVW_ <0.05/13 = 3.85 × 10^−3^ (8 smoking phenotypes‐schizophrenia reciprocal correlations, 4 smoking phenotypes‐antisocial behavior correlations, and 1 schizophrenia‐antisocial behavior correlation).

We calculated the *F* statistic to assess the strength of the instruments using the following equation:

$$F=\frac{R^{2}\times \left(N-2\right)}{1-R^{2}},$$

where *N* represents the sample size of the exposure GWAS data. *R* ^2^ is the proportion of the variance explained by each SNP, which was calculated using the following equation:

$$R^{2}=\frac{2\times \mathrm{MAF}\times \left(1-\mathrm{MAF}\right)\times \beta^{2}}{2\times \mathrm{MAF}\times \left(1-\mathrm{MAF}\right)\times \beta^{2}+2\times \mathrm{MAF}\times \left(1-\mathrm{MAF}\right)\times N\times \mathrm{se}{\left(\beta \right)}^{2}},$$

where MAF denotes the minor allele frequency of a given SNP, *β* denotes the effect size, *se*(*β*) denotes the standard error, and *N* represents the sample size of the GWAS data for the exposure.

If there are significant causal associations between smoking phenotypes and schizophrenia and both of them have causal effects on antisocial behavior, a two‐step MR analysis was performed to examine the mediation effects of schizophrenia/smoking phenotypes on the association between smoking phenotypes/schizophrenia and antisocial behavior. Specifically, in the first step, IVs for the exposure (smoking phenotypes/schizophrenia) were used to estimate the causal effect of the exposure on the potential mediators (schizophrenia/smoking phenotypes). In the second step, IVs for the identified mediators were used to assess the causal effect of the potential mediators on antisocial behavior. The “product of coefficients” method was utilized to evaluate the indirect effect of exposure on the outcome through each potential mediator, and the standard errors for the indirect effects were obtained using the delta method.^33^

### 2.4 Sensitivity analysis

To validate the robustness of our results, we performed a series of sensitivity analyses. First, to control for the potential confounding factors that may affect our results, we re‐performed MR analysis when controlling for the potential confounding factors. Davis et al. provided a summary of selected risk factors that were associated with schizophrenia.^34^ Moreover, previous studies have also identified determinants of antisocial behavior, such as drinking^35^ and childhood trauma.^36^ Hence, we evaluated the association of IVs with these known risk factors using large‐scale GWAS datasets, including the use of cannabis,^37^ childhood trauma,^38^ vitamin D,^39^ intelligence quotient,^40^ and drinking.^22^ Specifically, we removed the IVs associated with use of cannabis, childhood trauma, vitamin D, and intelligence quotient when conducting MR analysis for schizophrenia as the outcome variable. The IVs associated with childhood trauma and drinking were removed when conducting MR analysis for antisocial behavior as the outcome variable. To account for multiple testing, the significance level was Bonferroni‐corrected *p* < 0.05 [0.05/(The number of IVs × number of risk factors in each MR analysis)]. Second, we measured the heterogeneity among IVs using Cochran's *Q* statistic for IVW and Rücker's *Q* statistic for MR‐Egger. Third, we conducted MR‐Egger regression to assess potential directional horizontal pleiotropy, where an intercept term deviating from zero was considered as evidence of directional pleiotropic bias. Fourth, we performed the MR pleiotropy residual sum and outlier (MR‐PRESSO) test to identify possible horizontal pleiotropy. If pleiotropy was detected, the MR‐PRESSO outlier test was performed to identify outliers among the IVs and calculate MR estimates after removing them to eliminate the detected pleiotropy. Finally, we performed leave‐one‐out analyses to determine whether a single SNP was driving or biasing the estimate. The significance levels for the heterogeneity test, MR‐Egger intercept test, and MR‐PRESSO test were set at *p* < 0.05.

All the statistical analyses were conducted using the *TwoSampleMR* ^41^ (version 0.5.6), *mr.raps* ^30^ (version 0.2), *RadialMR* ^25^ (version 1.0), and *MRPRESSO* ^42^ (version 1.0) packages in *R*.

## 3 RESULTS

### 3.1 Bidirectional causal relationships of smoking phenotypes and schizophrenia

**FIGURE 1:**
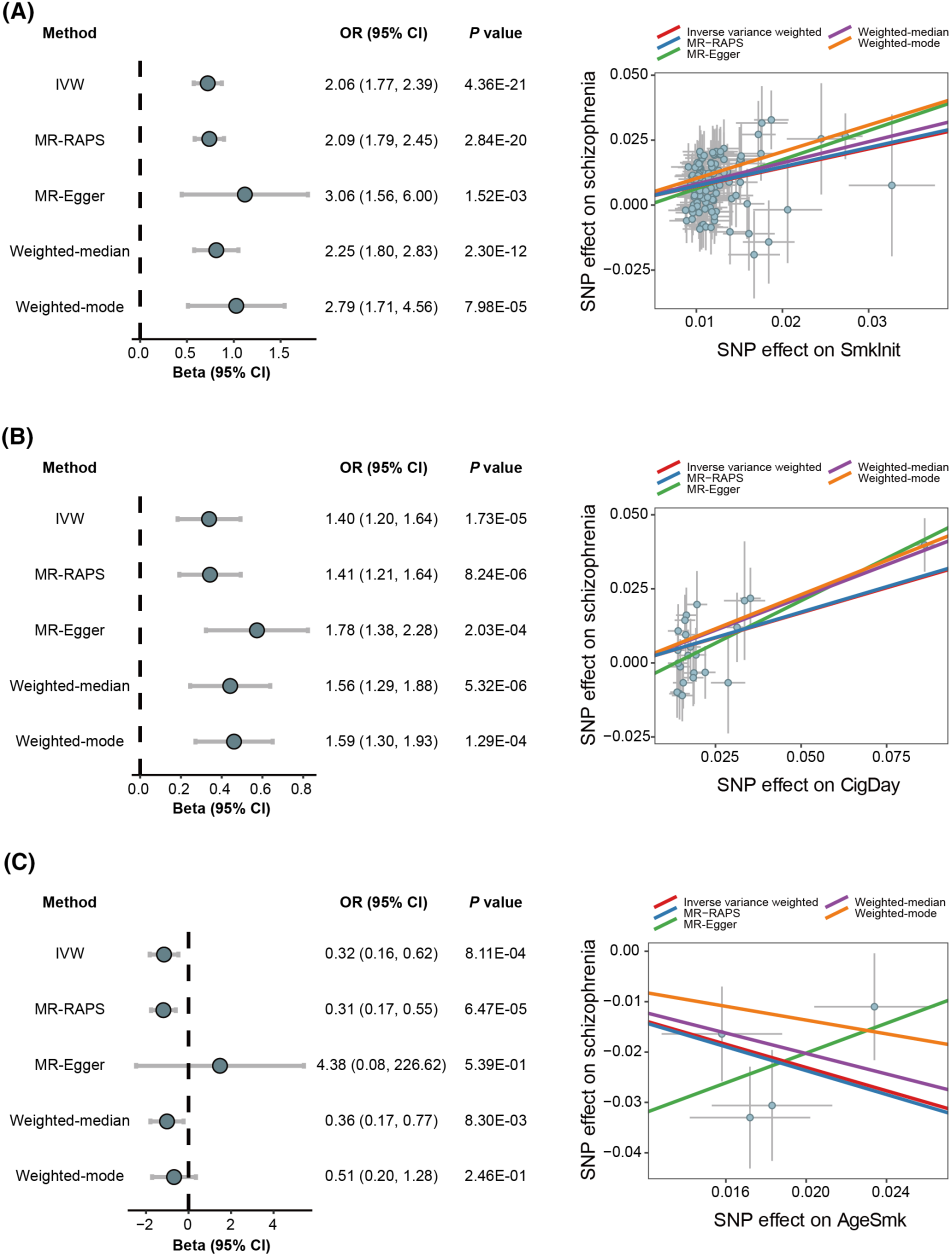
Two‐sample MR analyses of the causal effect of smoking phenotypes on schizophrenia. (A) MR results for the causal effect of SmkInit on schizophrenia (left). Scatter plots of the associations of SmkInit with schizophrenia (right). (B) MR results for the causal effect of CigDay on schizophrenia (left). Scatter plots of the associations of CigDay with schizophrenia (right). (C) MR results for the causal effect of AgeSmk on schizophrenia (left). Scatter plots of the associations of AgeSmk with schizophrenia (right). AgeSmk, age of smoking initiation; CI, confidence interval; CigDay, amount smoked; IVW, inverse variance weighted; MR‐RAPS: MR robust adjusted profile score; OR, odds ratio; SmkInit, smoking initiation; SNP, single nucleotide polymorphism.

We utilized 111 independent IVs that were genome‐wide and significantly associated with SmkInit at the threshold of *p* < 5 × 10^−8^ for MR analysis. The IVW method revealed that genetically predicted SmkInit was significantly associated with an increased risk of schizophrenia (odds ratio [OR] = 2.06, 95% confidence interval [CI] = 1.77–2.39, *p* = 4.36 × 10^−21^). Other MR methods yielded similar estimates of OR and also showed statistical significance (Figure 1A). For causality of SmkCes on schizophrenia, the IVW method showed that genetically predicted SmkCes was not significantly associated with schizophrenia with 11 IVs, and other MR methods provided similar estimates. The MR analysis was conducted by using 23 independent IVs that were related to CigDay. The IVW method showed that genetically predicted CigDay was significantly associated with an increased risk of schizophrenia (OR = 1.40, 95% CI = 1.20–1.64, *p* = 1.73 × 10^−5^). The causal effect across other MR methods was consistent (Figure 1B). Four IVs were employed to link AgeSmk with schizophrenia. The IVW method showed that genetically predicted younger AgeSmk was significantly associated with an increased risk of schizophrenia (OR = 0.32, 95% CI = 0.16–0.62, *p* = 8.11 × 10^−4^), and MR‐RAPS as well as weighted‐median methods yielded comparable results (Figure 1C). All the causal effects of smoking phenotypes on schizophrenia are shown in Supplementary Table S2. The *F* statistics of all IVs were larger than 10, indicating the absence of weak instrumental bias among these variables. The significant associations found above also remained when the confounding factors were removed (Supplementary Table S5).

We did not detect any significant causal effects of schizophrenia on smoking phenotypes. However, schizophrenia was nominally significantly associated with an earlier AgeSmk (OR = 0.99, 95% CI = 0.98–0.998, *p* = 0.02) using the IVW method. MR‐RAPS and weighted‐median methods provided similar estimates of OR and reached statistical nominal significance (MR‐RAPS: OR = 0.98, 95% CI = 0.97–0.996, *p* = 0.01; weighted‐median: OR = 0.99, 95% CI = 0.98–0.998, *p* = 0.02). Other MR methods provided similar estimates of OR but did not reach statistical significance. All the causal effects of schizophrenia on smoking phenotypes are shown in Supplementary Table S3, and all IVs exhibited *F* statistics greater than 10.

In leave‐one‐out sensitivity analyses, we found one genetic variant (rs17483721) might affect the MR estimates between CigDay and schizophrenia. The causal effect of CigDay on schizophrenia was significant at a trend level (OR_IVW_ = 1.23, 95% CI = 0.99–1.52, *p* = 0.06) after removing rs17483721. There were no influential outliers affecting the causal estimates of SmkInit and AgeSmk with schizophrenia (Figure S2) as well as the causal effects of schizophrenia on smoking phenotypes. The MR‐Egger intercept test indicated the presence of significant directional pleiotropy for the causal effect of CigDay on schizophrenia (*p* = 0.03). All the causal association analyses revealed no heterogeneity among IVs detected and MR‐PRESSO did not detect any outliers (all *p*s >0.05). The scatter plots illustrating the significant causal relationships between smoking phenotypes and schizophrenia are shown in Figure 1.

### 3.2 Causal effects of smoking phenotypes/schizophrenia on antisocial behavior

**FIGURE 2:**
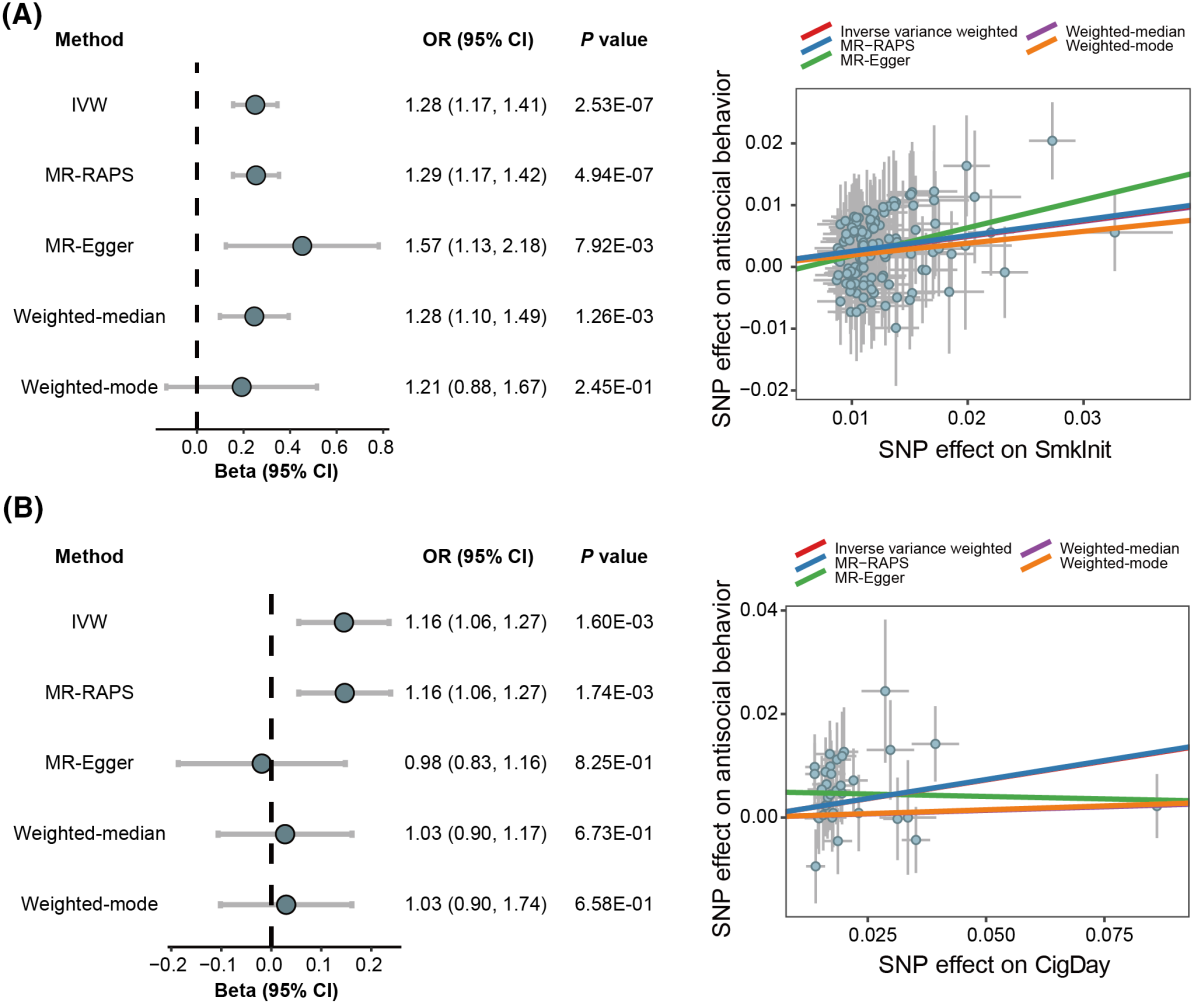
Two‐sample MR analyses of the causal effect of smoking phenotypes on antisocial behavior. (A) MR results for the causal effect of SmkInit on antisocial behavior (left). Scatter plots of the associations of SmkInit with antisocial behavior (right). (B) MR results for the causal effect of CigDay on antisocial behavior (left). Scatter plots of the associations of CigDay with antisocial behavior (right). CI, confidence interval; CigDay, amount smoked; IVW, inverse variance weighted; MR‐RAPS: MR robust adjusted profile score; OR, odds ratio; SmkInit, smoking initiation; SNP, single nucleotide polymorphism.

As shown in Figure 2 and Supplementary Table S4, we observed that two smoking phenotypes and schizophrenia were causally associated with antisocial behavior. The IVW method showed that genetically predicted SmkInit was significantly associated with an increased risk of antisocial behavior (OR = 1.28, 95% CI = 1.17–1.41, *p* = 2.53 × 10^−7^) using 125 independent IVs. Other MR methods provided similar estimates of OR and reached the statistical significance threshold of *p* < 0.05 except for the weighted‐mode method (Figure 2A). We used 34 independent IVs associated with CigDay for MR analysis, and the IVW method showed that genetically predicted CigDay was significantly associated with an increased risk of antisocial behavior (OR = 1.16, 95% CI = 1.06–1.27, *p* = 1.60 × 10^−3^). Only the MR‐RAPS method reached statistical significance (OR = 1.16, 95% CI = 1.06–1.27, *p* = 1.74 × 10^−3^) (Figure 2B). The IVW method showed that heavier SmkCes and younger AgeSmk were nominally significantly associated with an increased risk of antisocial behavior (SmkCes: OR = 1.28, 95% CI = 1.06–1.54, *p* = 9.50 × 10^−3^; AgeSmk: OR = 0.68, 95% CI = 0.49–0.93, *p* = 0.02).

The IVW, MR‐RAPS, and weighted‐median methods supported the nominally significant causal association between schizophrenia and antisocial behavior (IVW: OR = 1.02, 95% CI = 1.01–1.04, *p* = 7.12 × 10^−3^; MR‐RAPS: OR = 1.02, 95% CI = 1.01–1.04, *p* = 7.94 × 10^−3^; weighted‐median: OR = 1.03, 95% CI = 1.01–1.06, *p* = 0.01). However, the MR‐Egger and weighted‐mode methods did not reach statistical significance for the causal association. The absence of weak instrumental bias among the variables was indicated by *F* statistics greater than 10. The association between SmkInit and antisocial behavior remained significant when confounding factors were removed. Although the association between CigDay and antisocial behavior did not survive correction for multiple comparisons, it still exhibited nominal significance (Supplementary Table S5). In leave‐one‐out sensitivity analyses, no genetic variants could significantly affect the MR estimates (Figure S3). The MR‐Egger intercept test suggested that there was a significant directional pleiotropy for the causal effect of CigDay on antisocial behavior (*p* = 0.03). No significant heterogeneity among IVs was detected and MR‐PRESSO did not identify any outliers (all *p*s >0.05).

### 3.3 Mediation analysis

**FIGURE 3:**
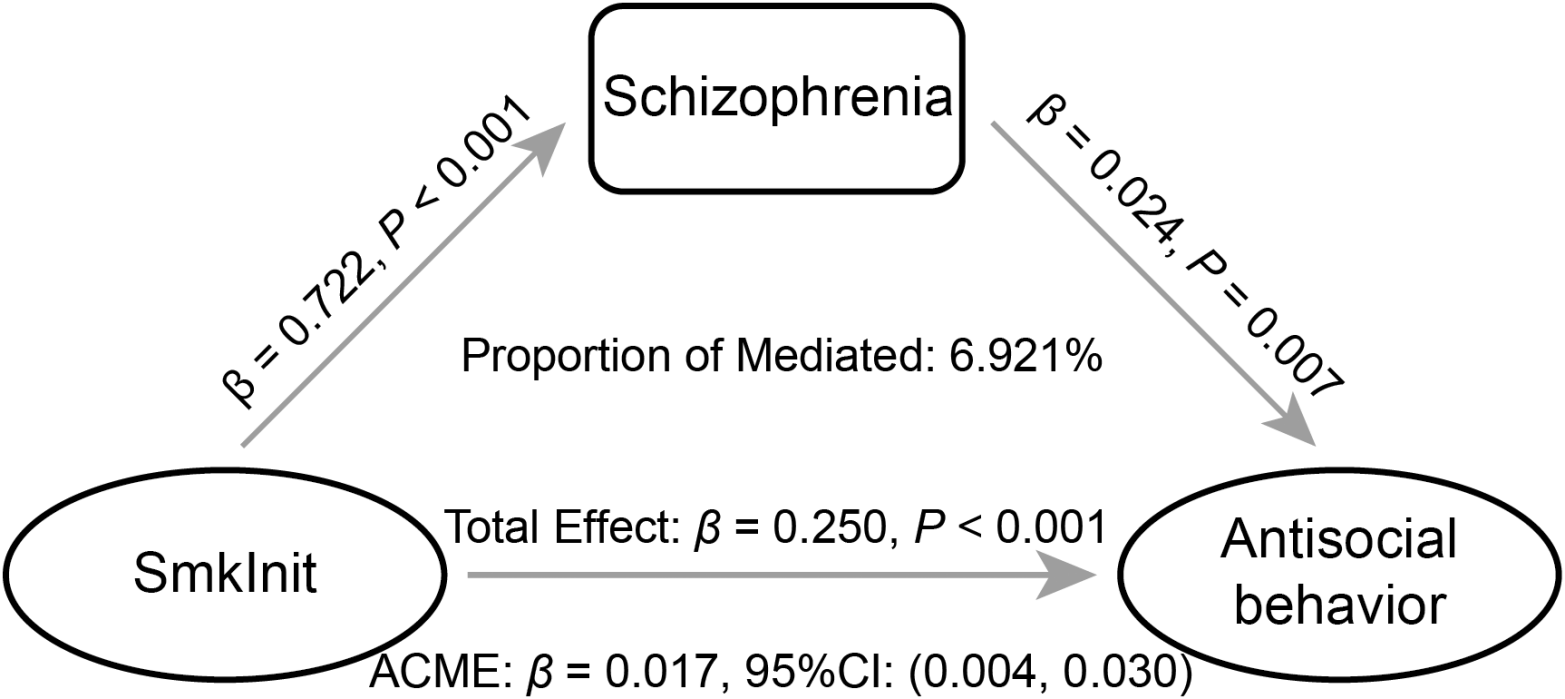
Mediation analysis of the causal effect of SmkInit on antisocial behavior via schizophrenia. ACME, average causal mediation effects; CI, confidence interval; SmkInit, smoking initiation.

Given that significant causal effects of SmkInit on schizophrenia and antisocial behavior were found in the above‐mentioned analyses, a two‐step MR analysis was performed to estimate the mediation effects of schizophrenia between SmkInit and antisocial behavior. The results revealed a significant mediating effect (*β* = 0.02, 95% CI = 0.004–0.03, *p* = 9.66 × 10^−3^) with a mediated proportion of 6.92% (Figure 3).

## 4 DISCUSSION

In this study, we utilized the MR method to examine the causal relationships among smoking phenotypes, schizophrenia, and antisocial behavior. We found that smoking phenotypes have a causal effect on the risk of schizophrenia and antisocial behavior. Our results were largely robust across various MR methods, which assume different horizontal pleiotropy,^43^ indicating that horizontal pleiotropy is unlikely to be an adequate explanation for our results. Moreover, we also conducted a mediation analysis to test potential pathways from smoking phenotypes/schizophrenia to antisocial behavior and showed for the first time that the effect of SmkInit on antisocial behavior risk was partially mediated by schizophrenia.

Our study provided support for a causal role of smoking initiation on liability to develop schizophrenia, which is in line with previous findings.^19^, ^20^, ^21^ Evidence suggests that there is a shared genetic component between smoking behaviors and schizophrenia,^44^ supporting the idea that there are common genetic components that contribute to the risk of both schizophrenia and the age of smoking initiation. Although previous studies have demonstrated the effects of early age of smoking initiation on schizophrenia,^7^, ^45^ these findings did not establish causation or the direction of causation. To our knowledge, our study is the first to report the causal effects of age of smoking initiation on schizophrenia. Earlier age of initiation smoking indicates longer tobacco exposure, demonstrating a clear dose–response relationship.^46^ The current result not only supported the causal role of smoking initiation in the development of schizophrenia but also provided evidence for the causal effects of initiation timing on the risk of developing schizophrenia. Longer nicotine exposure can result in long‐lasting alterations of dopaminergic and cholinergic pathways, leading to an increased risk of schizophrenia.^47^ Our findings demonstrated causal effect of CigDay on schizophrenia, indicating a possible dose–response relationship for both smoking severity and early age of smoking initiation represents more serious tobacco exposure. In addition, a nominal significant causal effect of schizophrenia risk on AgeSmk was found in this study. Our findings did not support any causal effects of schizophrenia risk on other smoking phenotypes, which is consistent with previous evidence that smoking initiation usually precedes psychotic experiences or schizophrenia.^48^

Previous studies have reported an association between smoke exposure and antisocial behavior.^15^, ^49^ In most individuals with schizophrenia, symptoms generally start after puberty, while patients with schizophrenia tend to have smoked for some years prior to the onset of the condition most patients with schizophrenia were exposed to tobacco during adolescence or even earlier. Given that smoking is prohibited in educational institutions, the act of smoking among adolescents can be considered a violation of rules, thereby constituting a facet of antisocial behavior. It has been also established that smoking is an independent predictor of hazardous conduct, which is another aspect of antisocial behavior.^50^ These factors may serve as underlying mechanisms for the co‐occurrence of smoking and antisocial behavior.

The most important finding of our study is that we provided evidence for a causal pathway of SmkInit and schizophrenia on antisocial behavior. Despite efforts to identify biological or genetic markers associated with aggression in schizophrenia, consistent findings to explain violent and aggressive behavior in schizophrenia have not been established. Comorbidity with substance abuse is a critical clinical indicator of increased aggressive behaviors and crime rates in patients with schizophrenia.^51^ A reduction in dopamine D2 receptor binding has been observed in those who suffer from tobacco addiction,^52^ and a corresponding decrease in dopamine D2 receptor function has been linked to an increase in impulsive tendencies.^53^ The results of our study suggest that smoking may lead to a decrease in dopamine D2 receptors, which in turn could contribute to an increase in antisocial behaviors. It is notable that schizophrenia may play a significant role in this process, as excessive dopamine receptors in specific brain regions have been linked to the development of this disorder.^54^

This study has several limitations. First, all the GWAS datasets used in this study are European ancestry samples. Thus, it should be cautious to generalize our findings to other ethnic groups. Second, the MR‐Egger intercept test indicated significant directional pleiotropy for the causal effect of CigDay on schizophrenia and antisocial behavior, which suggests that the results should be interpreted carefully. Third, despite employing procedures such as the MR‐Egger method, MR–PRESSO test, and conducting a manual search for confounding factors, the confounding bias may not be entirely ruled out. Finally, further efforts to elucidate the mechanisms underlying the association of the causal pathway are needed.

In conclusion, this study utilized the largest available GWAS summary statistics to investigate the causal pathway of smoking behaviors, schizophrenia, and antisocial behavior in European ancestry samples. The large sample sizes of GWAS, and homogeneous population structure, combined with various MR methods and a series of sensitivity analyses make the causal associations reliable. Hence, the aforementioned findings provide corroborative evidence that smoking is a hazardous element for schizophrenia, which can amplify antisocial behavior, emphasizing the potential for early intervention.

## AUTHOR CONTRIBUTIONS

Feng Liu, Lining Guo, and Jie Tang developed and designed the study concept. Minghui Zhang, Lining Guo, and Wei Li conducted the analysis. Kaizhong Xue, Zirui Wang, Yayuan Chen, Qiang Xu, Dan Zhu, Mengjing Cai, Juanwei Ma, Jia Yao, Yijing Zhang, and He Wang provided guidance for this study. Minghui Zhang wrote the first draft of this study. All the authors have approved the final article.

## CONFLICT OF INTEREST STATEMENT

The authors declare no conflict of interest.

## Supporting information


Data S1.

## Data Availability

The GWAS summary statistics utilized in this study can be downloaded by qualified researchers as they are accessible to the public.
