# Supplementary material for: Schizophrenia mediating the effect of smoking phenotypes on antisocial behavior: A Mendelian randomization analysis
Source: CNS Neurosci Ther. 2023 Aug 31;30(3):e14430. doi: 10.1111/cns.14430 (PMC10915990; doi:10.1111/cns.14430)
Supplement: Supplementary file 1 — Data S1. [file CNS-30-e14430-s001.docx]

**Supplementary Materials**

**Schizophrenia mediating the effect of smoking phenotypes on antisocial behavior: a Mendelian randomization analysis**

**Supplementary Tables**

| **Supplementary Table 1. Sources and description of GWAS summary statistics.** | | | |
| --- | --- | --- | --- |
| Phenotypes | Consortium | Sample size (cases) | Web sites for downloading |
| SmkInit^1^ | ALSPAC, ARIC - TOPMed, BEAGESS, BLS, CADD, deCODE, EGCUT, eMERGE - Cataracts, eMERGE - PAD, FinnTwin 1, FinnTwin 2, Framingham - TOPMed, Genes for Good, Harvard - Affy, Harvard - Illumina, Harvard - HumanCore, Harvard - OmniExpress, Harvard - OncoArray, HRS, HUNT, MCTFR, MESA - TOPMed, METSIM, NESCOG, NAG-FIN, NTR, SardiNIA, UKB, NINDS SiGN, FINRISK, WLS, Spit for Science, AMISH - TOPMed, GeneSTAR, GOLDN - TOPMed, IPF - TOPMed, CHS - TOPMed, HVH - TOPMed, JHS - TOPMed, VTE - TOPMed, WGHS - TOPMed, WHI - TOPMed | 805,431 | https://doi.org/10.13020/przg-dp88 |
| SmkCes^1^ | ALSPAC, ARIC - TOPMed, BEAGESS, BLS, CADD, COGEND, COPDGene - TOPMed, deCODE, EGCUT, eMERGE - Cataracts, eMERGE - PAD, FinnTwin 1, FinnTwin 2, Framingham - TOPMed, Genes for Good, Harvard - Affy, Harvard - Illumina, Harvard - HumanCore, Harvard - OmniExpress, Harvard - OncoArray, HUNT, MCTFR, METSIM, NESCOG, NAG-FIN, NTR, SardiNIA, UKB, NINDS SiGN, FINRISK, WLS, AMISH - TOPMed, CFS - TOPMed, ECLIPSE - TOPMed, GeneSTAR, GOLDN - TOPMed, Boston - TOPMed, IPF - TOPMed, CHS - TOPMed, HCHS SOL - TOPMed, HVH - TOPMed, VTE - TOPMed, WGHS - TOPMed, WHI - TOPMed | 388,313 | https://doi.org/10.13020/przg-dp88 |
| CigDay^1^ | ALSPAC, ARIC - TOPMed, BLS, CADD, COGEND, COPDGene - TOPMed, deCODE, EGCUT, FinnTwin 1, FinnTwin 2, Framingham - TOPMed, Genes for Good, Harvard - Affy, Harvard - Illumina, Harvard - HumanCore, Harvard - OmniExpress, Harvard - OncoArray, HRS, HUNT, MCTFR, MESA - TOPMed, METSIM, NESCOG, NAG-FIN, NTR, PAGE - CARDIA, QIMR, SardiNIA, UKB, FINRISK, WLS, Spit for Science, AMISH - TOPMed, CFS - TOPMed, ECLIPSE - TOPMed, GeneSTAR, GOLDN - TOPMed, Boston - TOPMed, IPF - TOPMed, CHS - TOPMed, HCHS SOL - TOPMed, WHI - TOPMed | 326,497 | https://doi.org/10.13020/przg-dp88 |
| AgeSmk^1^ | ALSPAC, ARIC - TOPMed, BLS, CADD, COGEND, COPDGene - TOPMed, deCODE, EGCUT, FinnTwin 1, FinnTwin 2, Framingham - TOPMed, Genes for Good, Harvard - Affy, Harvard - Illumina, Harvard - HumanCore, Harvard - OmniExpress, Harvard - OncoArray, HRS, HUNT, METSIM, NAG-FIN, NTR, QIMR, SardiNIA, UKB, FINRISK, WLS, AMISH - TOPMed, CFS - TOPMed, ECLIPSE - TOPMed, GeneSTAR, GOLDN - TOPMed, IPF - TOPMed, WHI - TOPMed | 323,386 | https://doi.org/10.13020/przg-dp88 |
| Schizophrenia^2^ | PGC | 130,644 (53,386) | https://figshare.com/articles/dataset/scz2022/19426775 |
| Antisocial behavior^3^ | ADH, ALSPAC, BIG, COGA, CoLaus, FinnTwin, GSA, iPSYCH, MCTFR, PAGES, PGC cohorts, QIMR - 1 (16UP, 25UP, GHA, PISA), QIMR - 2 (SS2), QIMR - 3 (GA), QIMR - 4 (T89), QIMR - 5 (NC), S4S, TEDS - 1 (Affymetrix), TEDS - 2 (OEE), TRAILS, DunedinM, E-Risk, PNC, QLSCD, QNTS | 85,359 | <https://ctg.cncr.nl/software/summary_statistics> |
| Abbreviations: AgeSmk, age of smoking initiation; CigDay, amount smoked; GWAS, genome-wide association studies; SmkCes, smoking cessation; SmkInit, smoking initiation. Referemces: 1. Saunders GRB, Wang X, Chen F, et al. Genetic diversity fuels gene discovery for tobacco and alcohol use. Nature. 2022;612(7941):720-724.  2. Trubetskoy V, Pardiñas AF, Qi T, et al. Mapping genomic loci implicates genes and synaptic biology in schizophrenia. Nature. 2022;604(7906):502-508.  3. Tielbeek JJ, Uffelmann E, Williams BS, et al. Uncovering the genetic architecture of broad antisocial behavior through a genome-wide association study meta-analysis. Mol Psychiatry. 2022;27(11):4453-4463. | | | |

**Supplementary Table 2. Results of the causal effect of smoking phenotypes on schizophrenia risk**

| **Smoking phenotypes** | **MR methods** | **Number of SNPs** | **OR** | **Lower CI** | **Upper CI** | ***P* value** |
| --- | --- | --- | --- | --- | --- | --- |
| SmkInit | IVW | 111 | 2.058 | 1.771 | 2.391 | 4.36E-21* |
| SmkInit | MR-RAPS | 111 | 2.093 | 1.789 | 2.449 | 2.84E-20* |
| SmkInit | MR-Egger | 111 | 3.060 | 1.560 | 6.002 | 1.52E-3* |
| SmkInit | Weighted-median | 111 | 2.255 | 1.797 | 2.8299 | 2.30E-12* |
| SmkInit | Weighted-mode | 111 | 2.791 | 1.708 | 4.560 | 7.98E-05* |
| SmkCes | IVW | 11 | 1.266 | 0.926 | 1.729 | 1.39E-01 |
| SmkCes | MR-RAPS | 11 | 1.269 | 0.922 | 1.747 | 1.43E-01 |
| SmkCes | MR-Egger | 11 | 0.924 | 0.441 | 1.935 | 8.39E-01 |
| SmkCes | Weighted-median | 11 | 1.099 | 0.736 | 1.641 | 6.44E-01 |
| SmkCes | Weighted-mode | 11 | 1.030 | 0.635 | 1.672 | 9.06E-01 |
| CigDay | IVW | 23 | 1.402 | 1.202 | 1.636 | 1.73E-05* |
| CigDay | MR-RAPS | 23 | 1.409 | 1.212 | 1.638 | 8.24E-06* |
| CigDay | MR-Egger | 23 | 1.774 | 1.381 | 2.278 | 2.03E-04* |
| CigDay | Weighted-median | 23 | 1.556 | 1.286 | 1.882 | 5.32E-06* |
| CigDay | Weighted-mode | 23 | 1.585 | 1.304 | 1.926 | 1.29E-04* |
| AgeSmk | IVW | 4 | 0.316 | 0.161 | 0.620 | 8.11E-04* |
| AgeSmk | MR-RAPS | 4 | 0.306 | 0.171 | 0.547 | 6.47E-05* |
| AgeSmk | MR-Egger | 4 | 4.382 | 0.085 | 226.623 | 5.39E-01 |
| AgeSmk | Weighted-median | 4 | 0.363 | 0.171 | 0.770 | 8.30E-03 |
| AgeSmk | Weighted-mode | 4 | 0.505 | 0.199 | 1.282 | 2.46E-01 |

Abbreviations: AgeSmk, age of smoking initiation; CI, confidence interval; CigDay, amount smoked; IVW, inverse variance weighted; MR-RAPS, MR robust adjusted profile score; OR, odds ratio; SmkCes, smoking cessation; SmkInit, smoking initiation; SNP, single nucleotide polymorphism.

Asterisks (*) indicates the level of statistical significance (Bonferroni correction, 0.05/13 = 3.85 × 10^-3^).

**Supplementary Table 3. Results of the causal effect of schizophrenia on smoking phenotypes**

| **Smoking phenotypes** | **MR methods** | **Number of SNPs** | **OR** | **Lower CI** | **Upper CI** | ***P* value** |
| --- | --- | --- | --- | --- | --- | --- |
| SmkInit | IVW | 50 | 1.008 | 0.999 | 1.017 | 6.45E-02 |
| SmkInit | MR-RAPS | 50 | 1.009 | 0.999 | 1.018 | 6.50E-02 |
| SmkInit | MR-Egger | 50 | 1.009 | 0.966 | 1.053 | 6.93E-01 |
| SmkInit | Weighted-median | 50 | 1.013 | 1.000 | 1.026 | 4.91E-02 |
| SmkInit | Weighted-mode | 50 | 1.021 | 0.991 | 1.052 | 1.85E-01 |
| SmkCes | IVW | 62 | 1.001 | 0.991 | 1.010 | 9.11E-01 |
| SmkCes | MR-RAPS | 62 | 1.001 | 0.991 | 1.010 | 9.11E-01 |
| SmkCes | MR-Egger | 62 | 0.991 | 0.943 | 1.040 | 7.03E-01 |
| SmkCes | Weighted-median | 62 | 0.998 | 0.985 | 1.012 | 8.20E-01 |
| SmkCes | Weighted-mode | 62 | 0.993 | 0.966 | 1.021 | 6.28E-01 |
| CigDay | IVW | 69 | 1.010 | 1.000 | 1.021 | 5.96E-02 |
| CigDay | MR-RAPS | 69 | 1.011 | 1.000 | 1.021 | 4.76E-02 |
| CigDay | MR-Egger | 69 | 1.032 | 0.970 | 1.097 | 3.22E-01 |
| CigDay | Weighted-median | 69 | 1.011 | 0.995 | 1.027 | 1.91E-01 |
| CigDay | Weighted-mode | 69 | 1.044 | 1.001 | 1.089 | 4.91E-02 |
| AgeSmk | IVW | 71 | 0.987 | 0.977 | 0.998 | 2.00E-02 |
| AgeSmk | MR-RAPS | 71 | 0.987 | 0.976 | 0.998 | 2.03E-02 |
| AgeSmk | MR-Egger | 71 | 0.989 | 0.936 | 1.044 | 6.82E-01 |
| AgeSmk | Weighted-median | 71 | 0.981 | 0.965 | 0.996 | 1.43E-02 |
| AgeSmk | Weighted-mode | 71 | 0.970 | 0.937 | 1.004 | 9.06E-02 |

Abbreviations: AgeSmk, age of smoking initiation; CI, confidence interval; CigDay, amount smoked; IVW, inverse variance weighted; MR-RAPS, MR robust adjusted profile score; OR, odds ratio; SmkCes, smoking cessation; SmkInit, smoking initiation; SNP, single nucleotide polymorphism.

**Supplementary Table 4. Results of the causal effects of of smoking phenotypes and schizophrenia on antisocial behavior**

| **Exposures** | **MR methods** | **Number of SNPs** | **OR** | **Lower CI** | **Upper CI** | ***P* value** |
| --- | --- | --- | --- | --- | --- | --- |
| SmkInit | IVW | 125 | 1.285 | 1.168 | 1.413 | 2.53E-07* |
| SmkInit | MR-RAPS | 125 | 1.289 | 1.168 | 1.423 | 4.94E-07* |
| SmkInit | MR-Egger | 125 | 1.572 | 1.132 | 2.183 | 7.92E-03 |
| SmkInit | Weighted-median | 125 | 1.279 | 1.101 | 1.486 | 1.26E-03* |
| SmkInit | Weighted-mode | 125 | 1.212 | 0.878 | 1.672 | 2.45E-01 |
| SmkCes | IVW | 17 | 1.280 | 1.062 | 1.542 | 9.50E-03 |
| SmkCes | MR-RAPS | 17 | 1.282 | 1.058 | 1.552 | 1.10E-02 |
| SmkCes | MR-Egger | 17 | 1.021 | 0.608 | 1.715 | 9.38E-01 |
| SmkCes | Weighted-median | 17 | 1.282 | 0.985 | 1.669 | 6.42E-02 |
| SmkCes | Weighted-mode | 17 | 1.256 | 0.903 | 1.746 | 1.94E-01 |
| CigDay | IVW | 34 | 1.156 | 1.057 | 1.265 | 1.60E-03* |
| CigDay | MR-RAPS | 34 | 1.158 | 1.056 | 1.270 | 1.74E-03* |
| CigDay | MR-Egger | 34 | 0.981 | 0.830 | 1.159 | 8.25E-01 |
| CigDay | Weighted-median | 34 | 1.028 | 0.904 | 1.168 | 6.73E-01 |
| CigDay | Weighted-mode | 34 | 1.030 | 0.904 | 1.174 | 6.58E-01 |
| AgeSmk | IVW | 6 | 0.677 | 0.492 | 0.930 | 1.62E-02 |
| AgeSmk | MR-RAPS | 6 | 0.674 | 0.482 | 0.943 | 2.12E-02 |
| AgeSmk | MR-Egger | 6 | 0.253 | 0.027 | 2.373 | 2.95E-01 |
| AgeSmk | Weighted-median | 6 | 0.630 | 0.425 | 0.936 | 2.20E-02 |
| AgeSmk | Weighted-mode | 6 | 0.596 | 0.346 | 1.028 | 1.22E-01 |
| Schizophrenia | IVW | 135 | 1.024 | 1.007 | 1.042 | 7.12E-03 |
| Schizophrenia | MR-RAPS | 135 | 1.025 | 1.006 | 1.043 | 7.94E-03 |
| Schizophrenia | MR-Egger | 135 | 1.033 | 0.967 | 1.104 | 3.39E-01 |
| Schizophrenia | Weighted-median | 135 | 1.034 | 1.007 | 1.061 | 1.32E-02 |
| Schizophrenia | Weighted-mode | 135 | 1.043 | 0.987 | 1.103 | 1.38E-01 |

Abbreviations: AgeSmk, age of smoking initiation; CI, confidence interval; CigDay, amount smoked; IVW, inverse variance weighted; MR-RAPS, MR robust adjusted profile score; OR, odds ratio; SmkCes, smoking cessation; SmkInit, smoking initiation; SNP, single nucleotide polymorphism.

Asterisks (*) indicates the level of statistical significance (Bonferroni correction, 0.05/13 = 3.85 × 10^-3^).

**Supplementary Table 5. Causal effect of smoking phenotypes on schizophrenia and antisocial behavior risk when controlling for the potential confounding factors**

| **Smoking phenotypes** | **Outcome** | **MR methods** | **Number of SNPs** | **OR** | **Lower CI** | **Upper CI** | ***P* value** |
| --- | --- | --- | --- | --- | --- | --- | --- |
| SmkInit | Schizophrenia | IVW | 93 | 1.989 | 1.678 | 2.358 | 2.47E-15* |
| SmkInit | Schizophrenia | MR-RAPS | 93 | 2.024 | 1.694 | 2.419 | 8.66E-15* |
| SmkInit | Schizophrenia | MR-Egger | 93 | 2.172 | 0.842 | 5.604 | 1.12E-01 |
| SmkInit | Schizophrenia | weighted-median | 93 | 2.062 | 1.600 | 2.656 | 2.15E-08* |
| SmkInit | Schizophrenia | weighted-mode | 93 | 3.375 | 1.664 | 6.845 | 1.09E-03* |
| CigDay | Schizophrenia | IVW | 18 | 1.392 | 1.177 | 1.646 | 1.11E-04* |
| CigDay | Schizophrenia | MR-RAPS | 18 | 1.398 | 1.192 | 1.639 | 3.87E-05* |
| CigDay | Schizophrenia | MR-Egger | 18 | 1.846 | 1.433 | 2.377 | 2.17E-04* |
| CigDay | Schizophrenia | weighted-median | 18 | 1.559 | 1.281 | 1.896 | 9.10E-06* |
| CigDay | Schizophrenia | weighted-mode | 18 | 1.588 | 1.306 | 1.930 | 2.34E-04* |
| AgeSmk | Schizophrenia | IVW | 18 | 1.588 | 1.306 | 1.930 | 2.34E-04* |
| AgeSmk | Schizophrenia | MR-RAPS | 3 | 0.294 | 0.154 | 0.564 | 2.28E-04* |
| AgeSmk | Schizophrenia | MR-Egger | 3 | 37.662 | 0.465 | 3050.362 | 3.52E-01 |
| AgeSmk | Schizophrenia | weighted-median | 3 | 0.292 | 0.141 | 0.607 | 9.70E-04* |
| AgeSmk | Schizophrenia | weighted-mode | 3 | 0.623 | 0.269 | 1.440 | 3.84E-01 |
| SmkInit | Antisocial behavior | IVW | 107 | 1.248 | 1.123 | 1.387 | 4.08E-05* |
| SmkInit | Antisocial behavior | MR-RAPS | 107 | 1.251 | 1.121 | 1.397 | 6.73E-05* |
| SmkInit | Antisocial behavior | MR-Egger | 107 | 1.294 | 0.884 | 1.896 | 1.88E-01 |
| SmkInit | Antisocial behavior | weighted-median | 107 | 1.273 | 1.081 | 1.498 | 3.77E-03* |
| SmkInit | Antisocial behavior | weighted-mode | 107 | 1.250 | 0.926 | 1.689 | 1.48E-01 |
| CigDay | Antisocial behavior | IVW | 30 | 1.134 | 1.032 | 1.246 | 9.15E-03 |
| CigDay | Antisocial behavior | MR-RAPS | 30 | 1.135 | 1.031 | 1.249 | 9.58E-03 |
| CigDay | Antisocial behavior | MR-Egger | 30 | 0.979 | 0.827 | 1.157 | 8.02E-01 |
| CigDay | Antisocial behavior | weighted-median | 30 | 1.027 | 0.903 | 1.169 | 6.84E-01 |
| CigDay | Antisocial behavior | weighted-mode | 30 | 1.028 | 0.896 | 1.180 | 6.92E-01 |

Abbreviations: AgeSmk, age of smoking initiation; CI, confidence interval; CigDay, amount smoked; IVW, inverse variance weighted; MR-RAPS, MR robust adjusted profile score; OR, odds ratio; SmkInit, smoking initiation; SNP, single nucleotide polymorphism.

Asterisks (*) indicates the level of statistical significance (Bonferroni correction, 0.05/13 = 3.85 × 10^-3^).

**
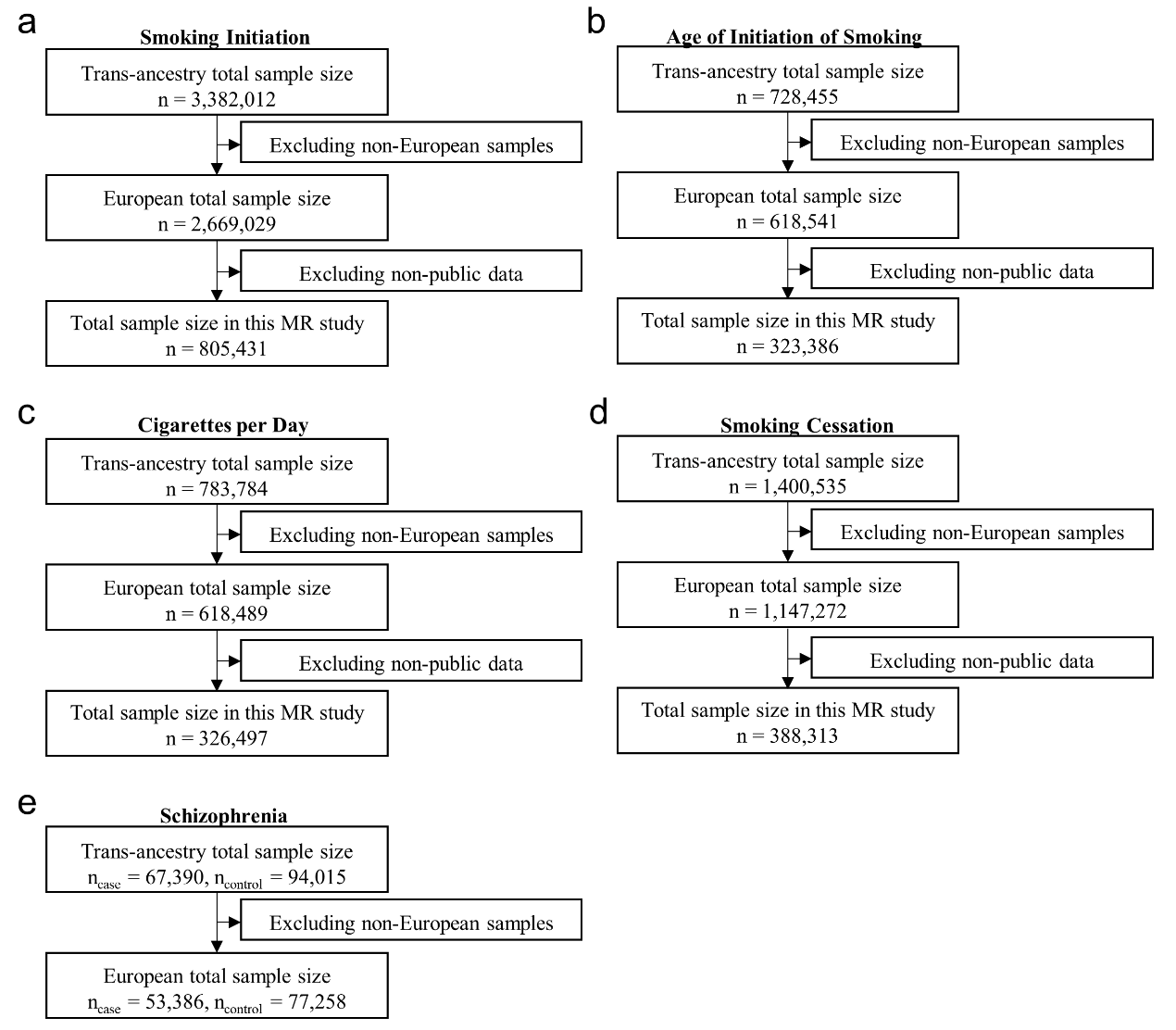
**

**Supplementary Fig. 1. Flow chart of participant selection in this MR study.**

Note: all samples (n = 85,359) were included in this MR study for antisocial behavior.

**
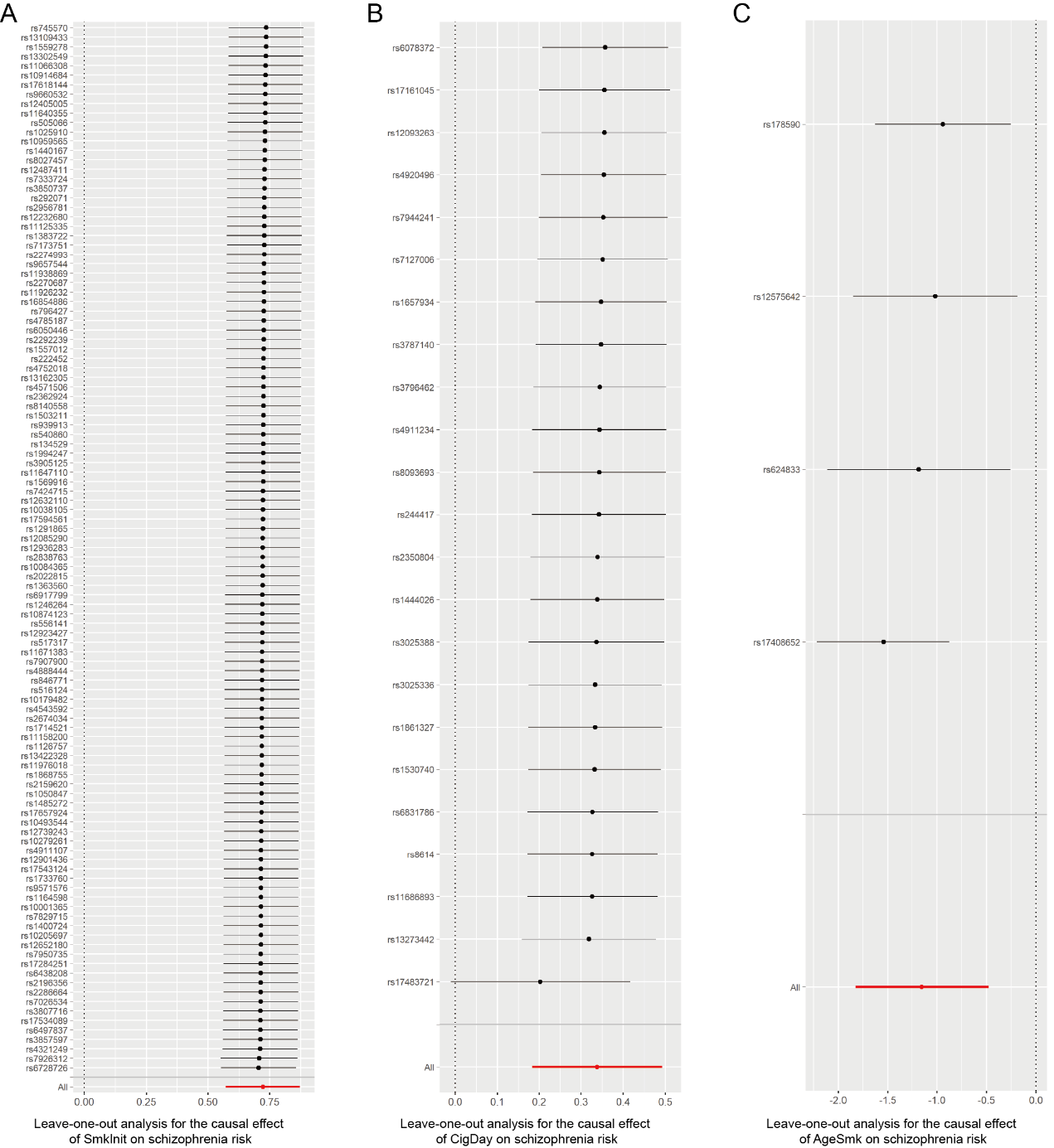
**

**Supplementary Fig. 2. Leave-one-out analysis MR analysis for the causal effect of smoking phenotypes on schizophrenia risk.** A, Leave-one-out analysis for the causal effect of SmkInit on schizophrenia risk. B, Leave-one-out analysis for the causal effect of CigDay on schizophrenia risk. C, Leave-one-out analysis for the causal effect of AgeSmk on schizophrenia risk. Abbreviations: AgeSmk, age of smoking initiation; CigDay, amount smoked; SmkInit, smoking initiation.

**
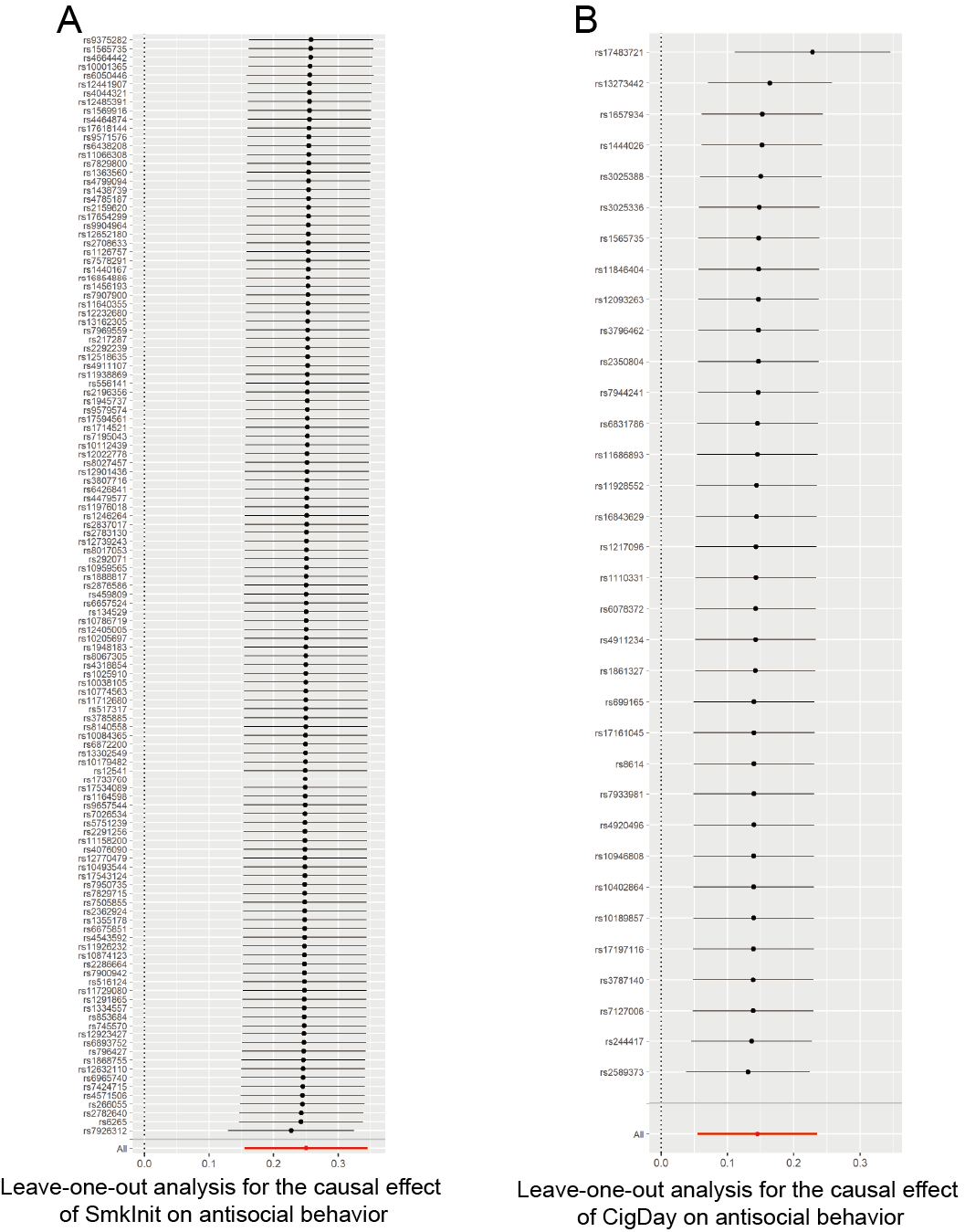
**

**Supplementary Fig. 3. Leave-one-out analysis MR analysis for the causal effect of smoking phenotypes on antisocial behavior.** A, Leave-one-out analysis for the causal effect of SmkInit on antisocial behavior. B, Leave-one-out analysis for the causal effect of CigDay on antisocial behavior. Abbreviations: CigDay, amount smoked; SmkInit, smoking initiation.
